# Supplementary material for: Induced cortical tension restores functional junctions in adhesion-defective carcinoma cells
Source: Nat Commun. 2017 Nov 28;8:1834. doi: 10.1038/s41467-017-01945-y (PMC5705652; doi:10.1038/s41467-017-01945-y)
Supplement: Supplementary file 1 — Supplementary Information [file 41467_2017_1945_MOESM1_ESM.pdf]

Supplementary Figure 1

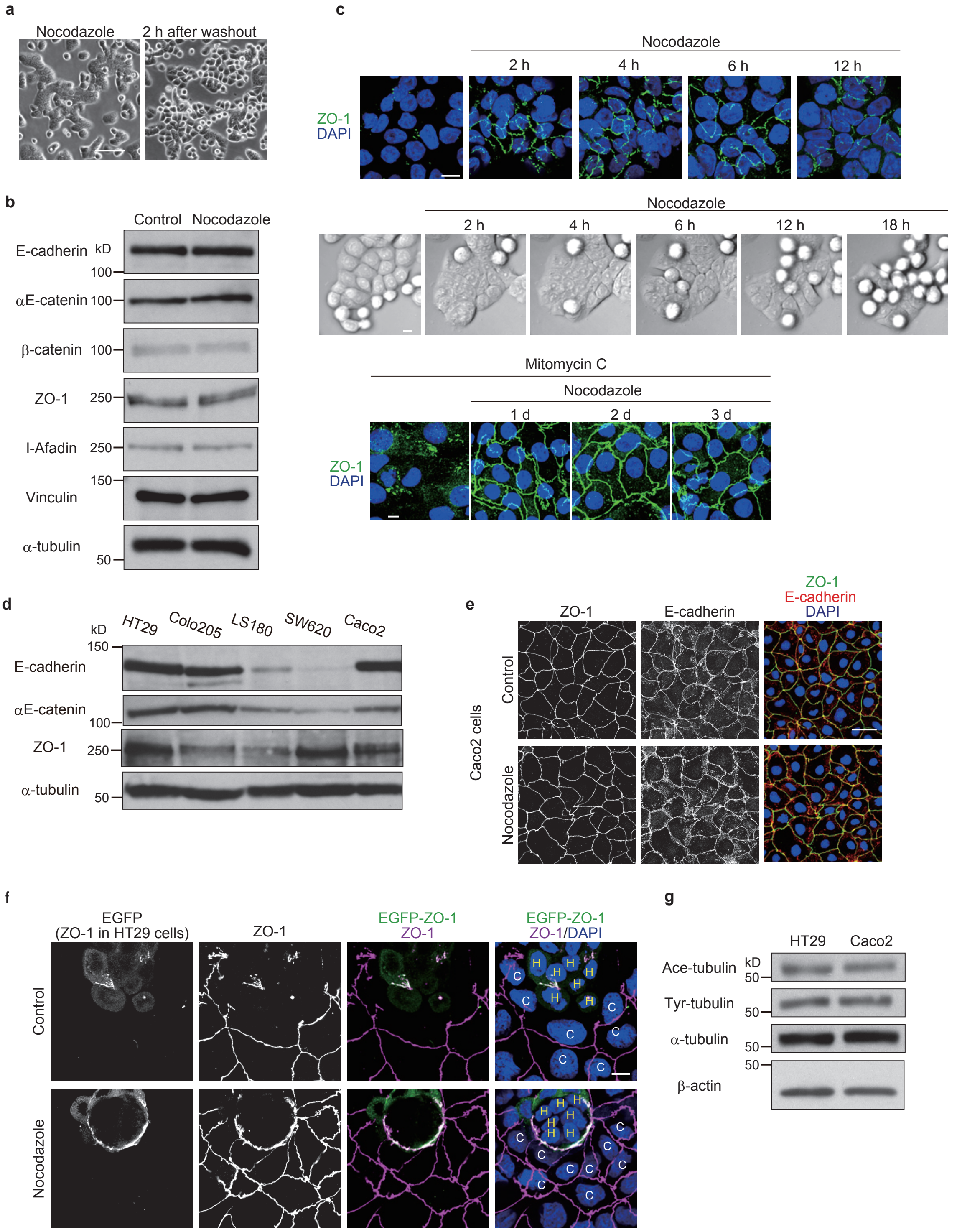

Supplementary Figure 1. Effects of nocodazole on cell junctions in colon carcinoma lines. (**a**) Effects of nocodazole washout. After treating with nocodazole for 1 hr, cells were washed out (right) or not (left), and cultured for another 2 hrs (right). (**b**) Western blots for E-cadherin, αE-catenin, β-catenin, ZO-1, l-afadin, vinculin, and α-tubulin in HT29 cells treated or not treated with nocodazole. (**c**) Persistence of nocodazole effects. *Upper panels*, HT29 cells were incubated with nocodazole for 2, 4, 6 and 12 hrs, and immunostaining for ZO-1. *Middle panels*, Time-lapse images of HT29 cells treated with nocodazole. Rounded cells are likely mitosis-arrested cells. *Lower panels*, HT29 cells were treated with 8 μg/ml mitomycin C for 2 hrs, then re-plated onto cover slides. After one day, cells were incubated with nocodazole for the indicated days, followed by immunostaining for ZO-1. Images in the middle panels are identical to the left-hand movie in Supplementary Video 2. (**d**) Western blots for E-cadherin, αE-catenin, ZO-1 and α-tubulin in HT29, Colo205, LS180, SW620, and Caco2 cells. We noted that the SW620 line was a mixture of E-cadherin–positive and –negative cells. Therefore, we selected E-cadherin–positive colonies for the observations shown in Fig. 1**g**. (**e**) Staining for ZO-1, E-cadherin and DNA in Caco2 cells treated with 10 μM nocodazole for 1 hr. (**f**) HT29 cells stably expressing EGFP–ZO-1 were cultured with Caco2 cells for 2 days. "H" and "C" indicate HT29 and Caco2 cells, respectively. ZO-1 is detectable at the boundaries between HT29 and Caco2 cells only after nocodazole treatment. ZO-1 localized between nocodazole-treated HT29 cells are out of focus in these images. (**g**) Analysis of microtubule posttranslational modification by Western blotting for acetylated (Ace)-tubulin, tyrosinated (Tyr)-tubulin, α-tubulin and β-actin. Scale bars, 100 μm (**a**), 10 μm (**c**, **f**), 50 μm (**e**).

Supplementary Figure 2

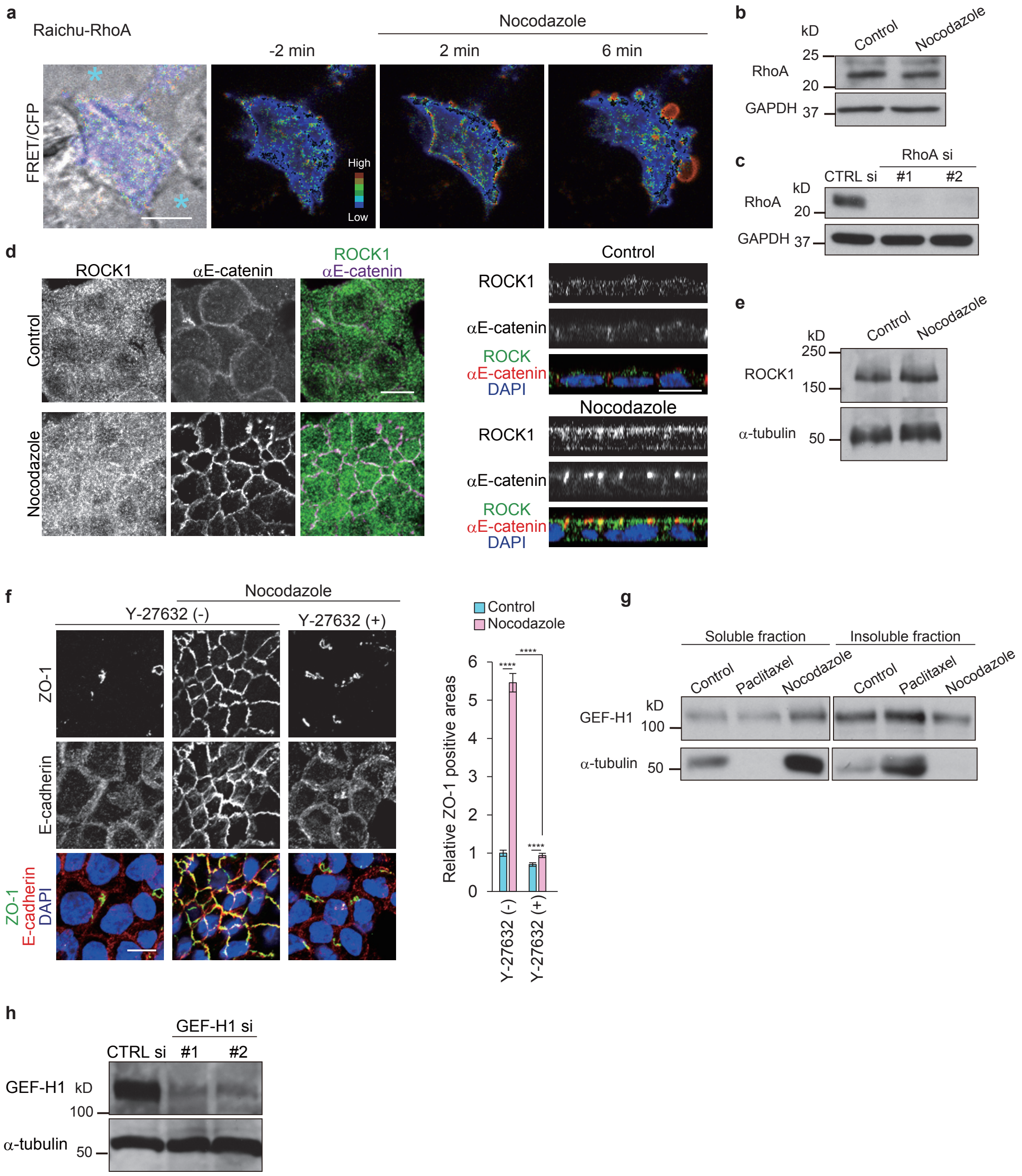

Supplementary Figure 2. Effects of nocodazole on RhoA or ROCK1, and knockdown efficiencies of various siRNAs. (**a**) Time-lapse images of a RhoA-specific Raichu probe expressed in HT29 cells, which were incubated with 20 μM nocodazole at the indicated times. The apical surface of the cells was focused. Red represents the sites of RhoA activation. Light blue asterisks show non-cellular spaces. (**b**) Western blots for RhoA and GAPDH in HT29 cells treated or not treated with nocodazole. (**c**) Knockdown efficiency of RhoA-specific siRNAs. (**d**) Immunostaining for ROCK1 and αE-catenin in HT29 cells. Lateral views of cells are also shown at the right. (**e**) Western blots for ROCK and GAPDH in HT29 cells treated or not treated with nocodazole. (**f**) Effects of Y-27632 on the distribution of ZO-1 and E-cadherin. Cells were treated with 10 μM Y-27632 for 2 hrs, and then with nocodazole for another one hr. Relative ZO-1 positive areas are also shown. n = 69, 73, 70 and 71 fields for control/Y-23632(-), nocodazole-treated/Y-23632(-), control/Y-23632(+) and nocodazole-treated/Y-23632(+) HT29 cultures, respectively, pooled from two independent experiments. ****$P < 0.0001$ by two-tailed Mann-Whitney U test. (**g**) Soluble and insoluble fractions of GEF-H1 or microtubules in HT29 cells, treated or not treated with 10 μM paclitaxel or 10 μM nocodazole. Soluble GEF-H1 increases in nocodazole-treated cells. (**h**) Knockdown efficiency of GEF-H1 specific siRNAs. Scale bars, 10 μm.

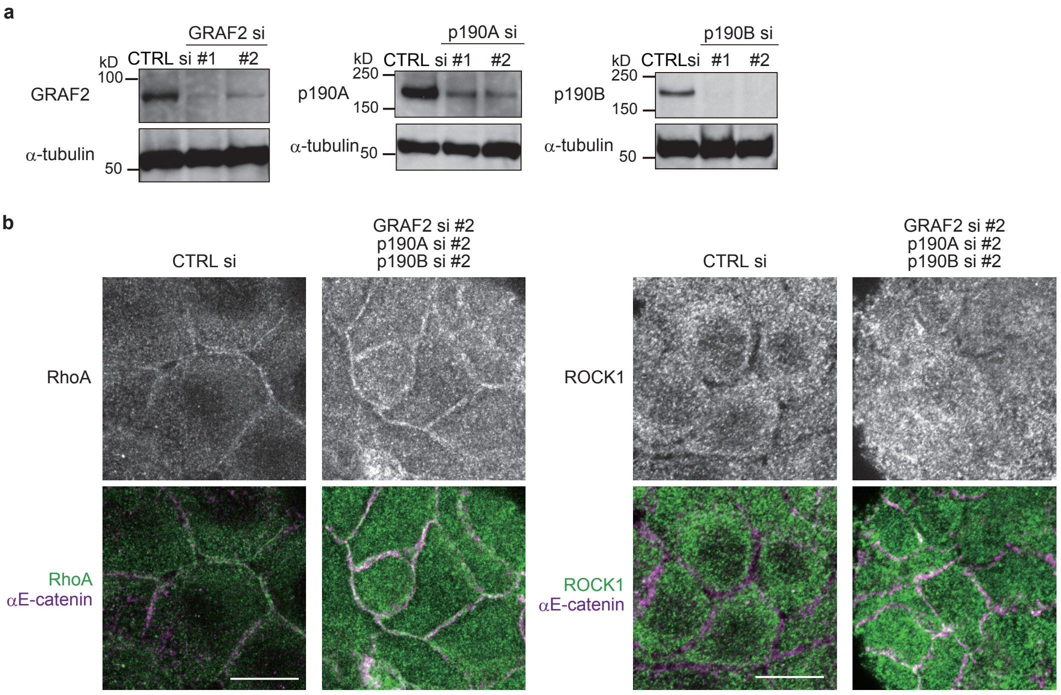

Supplementary Figure 3. Data on GRAF2, p190A and p190B knock down. (**a**) Knockdown efficiency of siRNAs specific for GRAF2, p190A and p190B. (**b**) Immunostaining for RhoA or ROCK and αE-catenin in HT29 cells transfected with CTRL siRNA or a mix of GRAF2 si #2, p190A si #2 and p190B si #2 (GAPs si #2). Scale bars, 10 µm.

Supplementary Figure 4

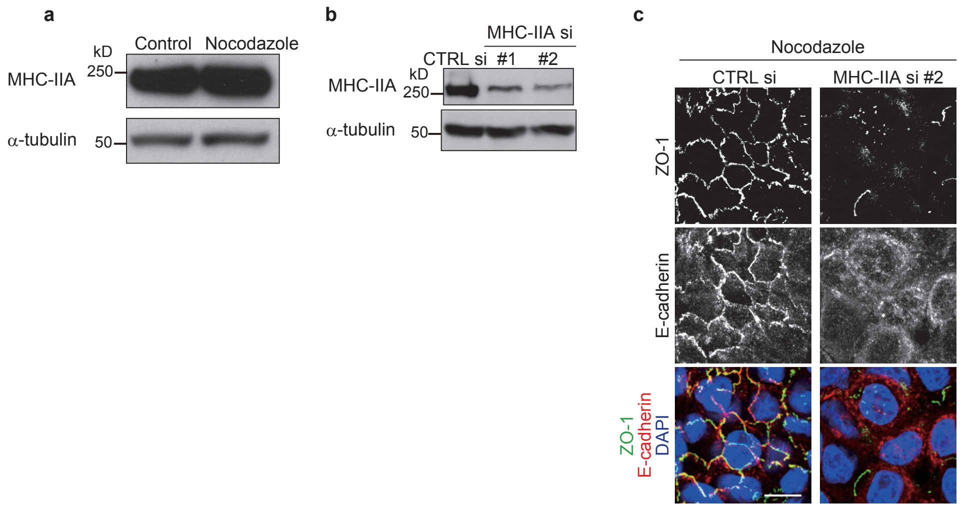

Supplementary Figure 4. Data on MHC-IIA. (**a**) Western blots for MHC-IIA in HT29 cells treated or not treated with nocodazole. (**b**) Knockdown efficiency of siRNAs specific for MHC-IIA. (**c**) Effects of MHC-IIA knockdown on junction formation. Scale bars, 10 μm.

Supplementary Figure 5

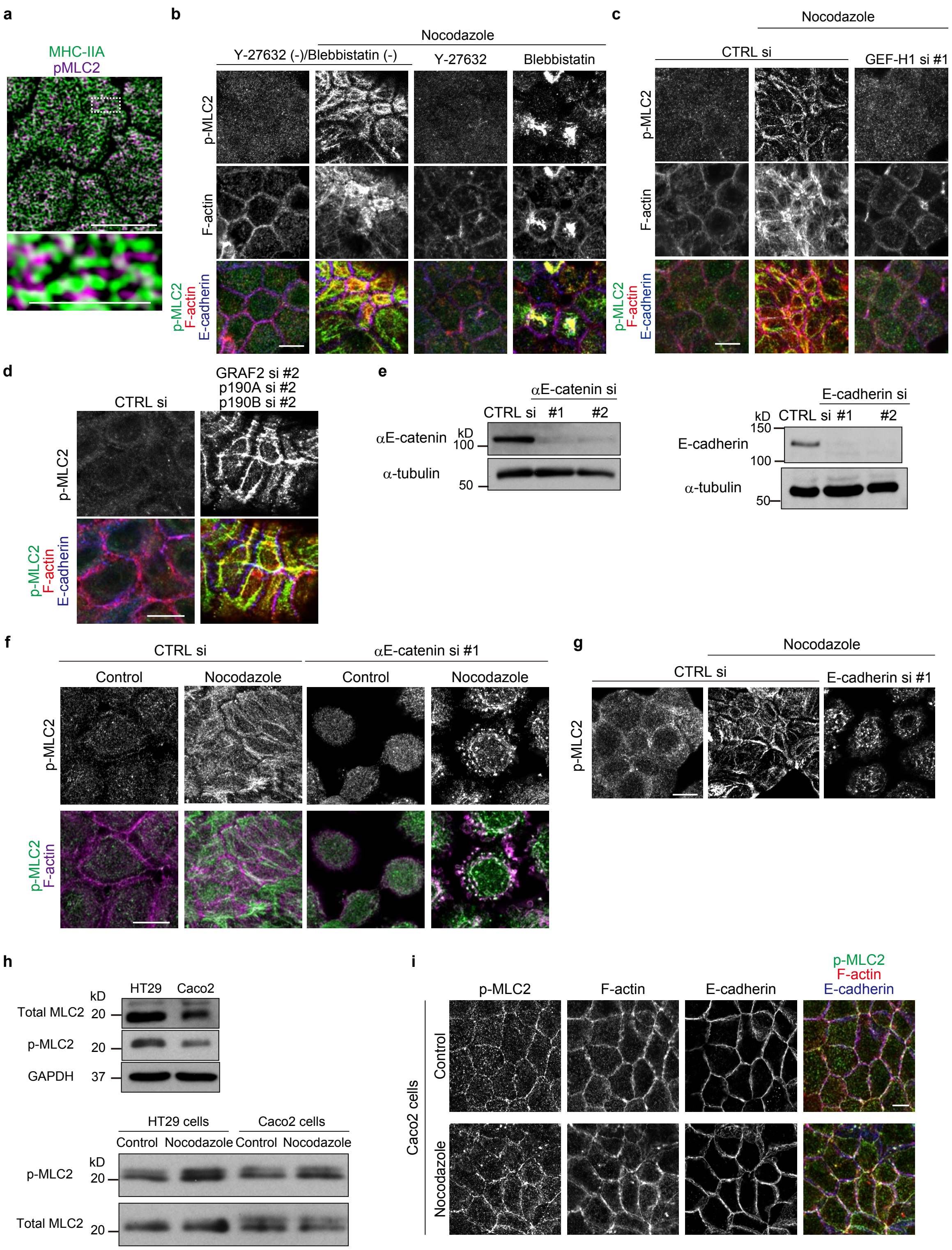

Supplementary Figure 5. p-MLC2 distribution in various conditions, and αE-catenin knockdown efficiency. (**a**) MHC-IIA and p-MLC in HT29 cells treated with nocodazole. Images were obtained with SIM. Boxed area is enlarged below. (**b**) Effects of Y-27632 and blebbistatin on the distribution of p-MLC2, F-actin and E-cadherin in HT29 cells. Cells were first treated with 10 μM Y-27632 or 100 μM blebbistatin for 2 hrs, and then with nocodazole for another one hr. (**c**) Effects of GEF-H1 knockdown on the distribution of p-MLC2, F-actin and E-cadherin in HT29 cells. (**d**) Effects of GAPs knockdown on the distribution of p-MLC2, F-actin and E-cadherin in HT29 cells. (**e**) Knockdown efficiency of αE-catenin and E-cadherin specific siRNAs. (**f**) Effects of αE-catenin knockdown on the distribution of p-MLC2 and F-actin in HT29 cells. (**g**) Effects of E-cadherin knockdown on the distribution of p-MLC2. (**h**) Immunoblotting detection of total MLC2 and p-MLC2 in HT29 or Caco2 cells (upper panel). Changes in p-MLC2 and MLC2 levels after nocodazole treatment are also shown (lower panel). (**i**) Immunostaining for p-MLC2, F-actin and E-cadherin in Caco2 cells. Scale bars, 10 μm.

Supplementary Figure 6

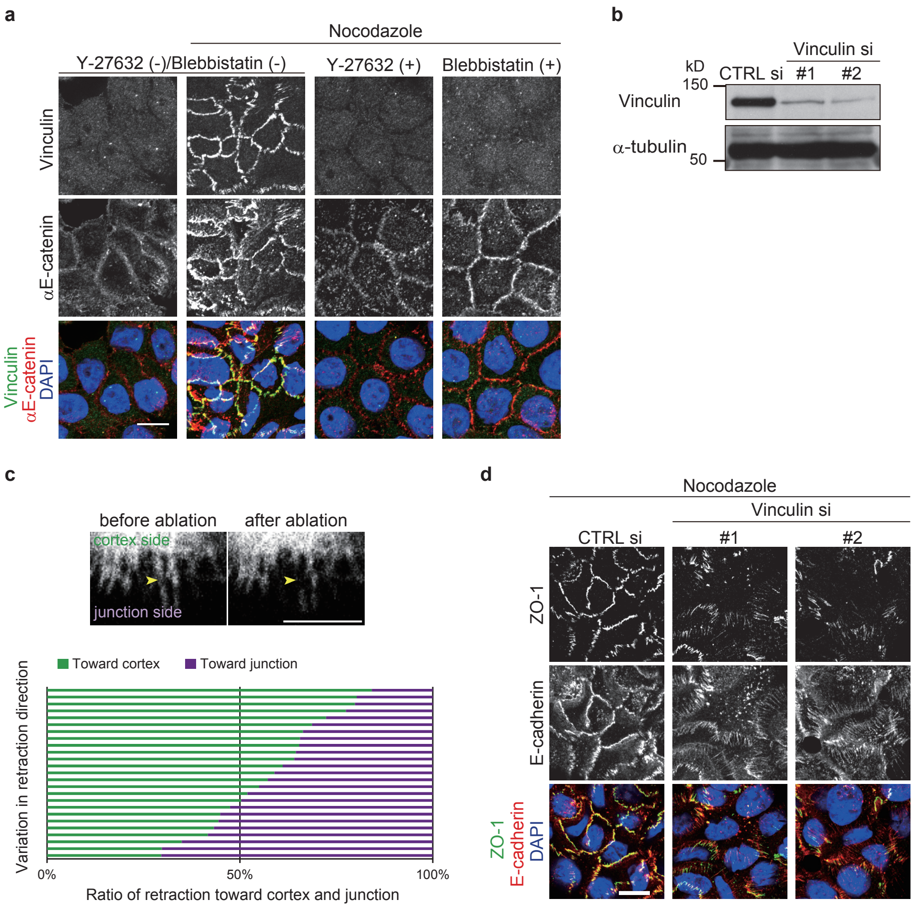

Supplementary Figure 6. Data on vinculin. (**a**) Effects of Y-27632 and blebbistatin on the distribution of vinculin or αE-catenin in HT29 cells. (**b**) Knockdown efficiency of siRNAs specific for vinculin. (**c**) Laser ablation of Lifeact-EGFP labeled protrusions in vinculin depleted HT29 cells treated with nocodazole. Yellow arrowheads indicate the site for laser application. n = 25 protrusions pooled from two independent experiments. Images are identical to Supplementary Video 7. (**d**) Effects of vinculin knockdown on the distribution of ZO-1 and E-cadherin in HT29 cells. Scale bars, 10 μm (**a**, **d**); 5 μm (**c**).

Supplementary Figure 7

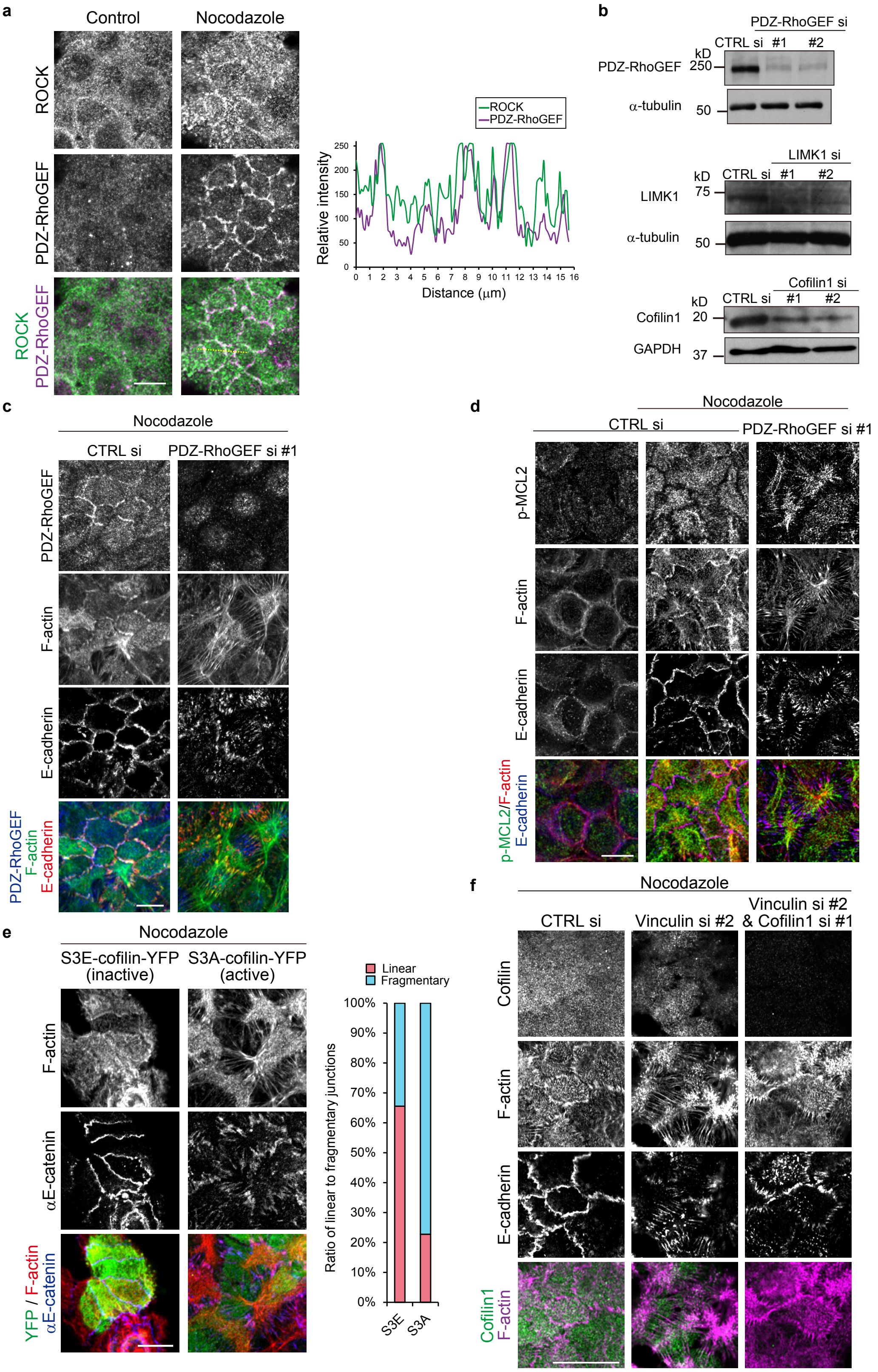

Supplementary Figure 7. Data on PDZ-RhoGEF, LIMK1 and cofilin. (**a**) Immunostaining for PDZ-RhoGEF and ROCK in HT29 cells. Relative intensity of PDZ-RhoGEF and ROCK signals were scanned along the yellow line. (**b**) Knockdown efficiency of siRNAs specific for PDZ-RhoGEF, LIMK1, and cofilin1. (**c**) Immunostaining for PDZ-RhoGEF, F-actin and E-cadherin in nocodazole-treated HT29 cells that were transfected with CTRL si or PDZ-RhoGEF si #1. (**d**) Effects of PDZ-RhoGEF knockdown on the distribution of p-MLC2, F-actin and E-cadherin in HT29 cells. (**e**) Effects of S3E- or S3A-cofilin tagged with YFP. The ratio of linear to fragmentary junctions was calculated. n = 1,228 junction in 101 colonies for S3E-cofilin and 926 junctions in 89 colonies for S3A-cofilin, pooled from two independent experiments. (**f**) Effects of vinculin/cofilin double knockdown on the distribution of F-actin and E-cadherin in HT29 cells. Images in **d** and **e** were obtained with Airyscan. Scale bars, 10 μm.

Supplementary Figure 8

Figure 2a

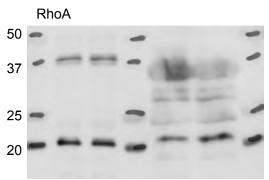

Figure 5a

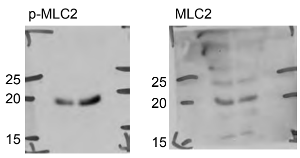

Supplementary Figure 1b

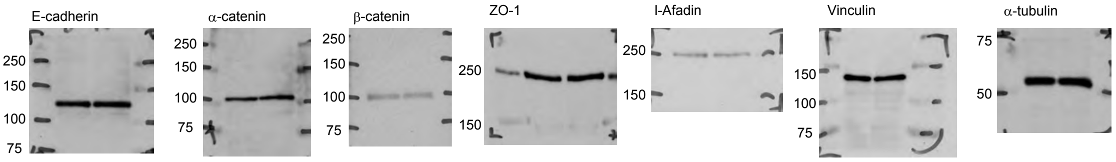

Supplementary Figure 1d

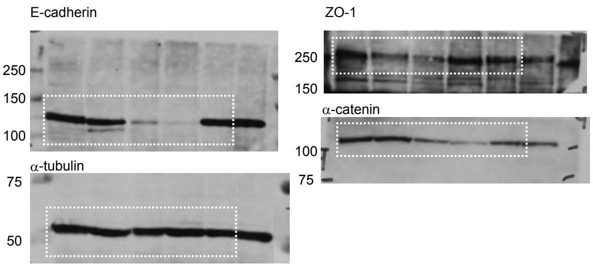

Supplementary Figure 1g

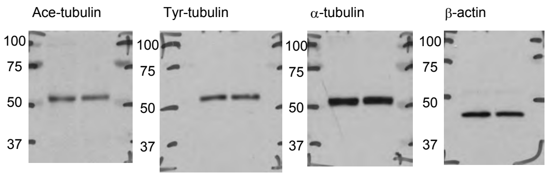

Supplementary Figure 2b

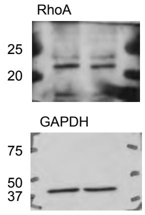

Supplementary Figure 2c

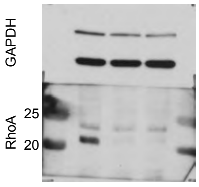

Supplementary Figure 2e

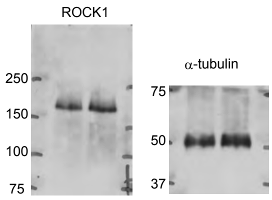

Supplementary Figure 2g

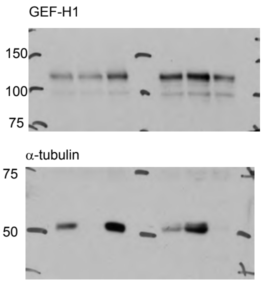

Supplementary Figure 2h

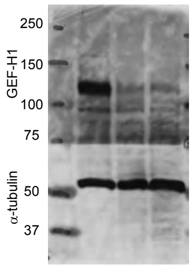

Supplementary Figure 3a

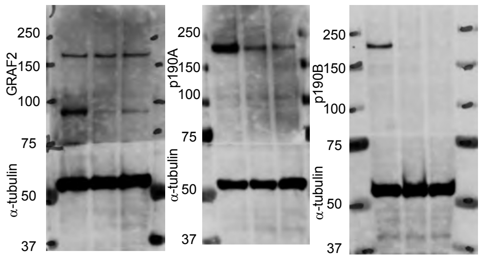

Supplementary Figure 4a

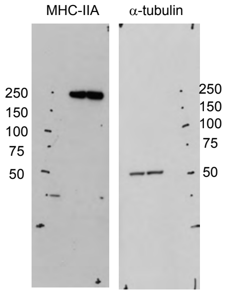

Supplementary Figure 4b

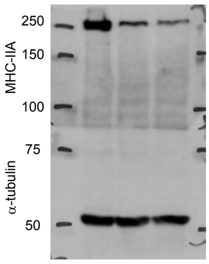

Supplementary Figure 8 Continued

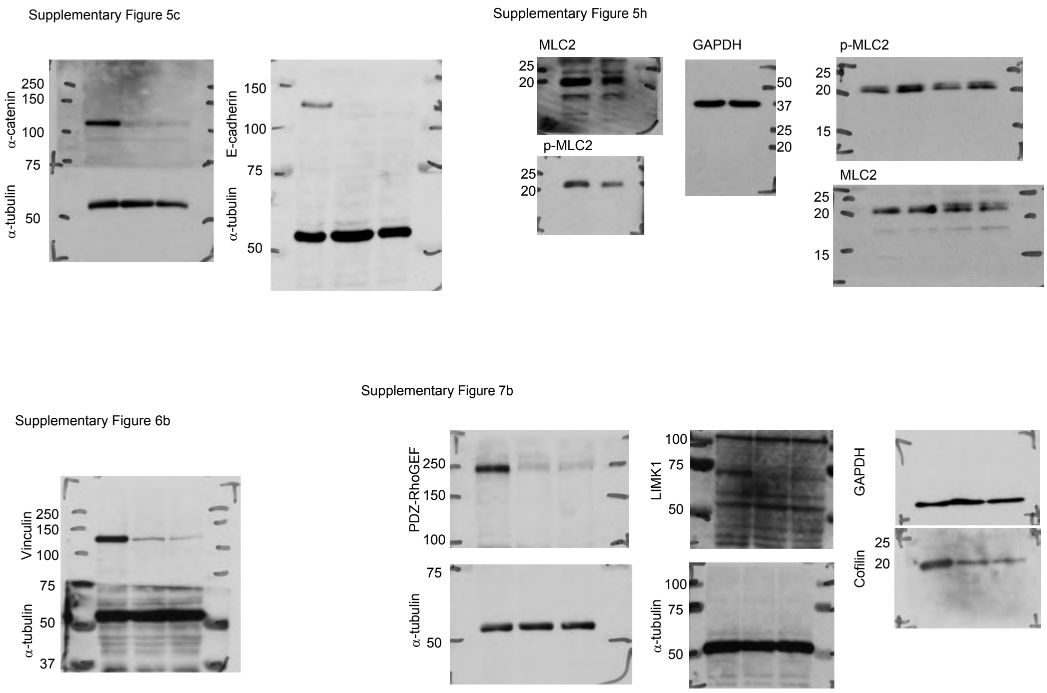

Supplementary Figure 8. Uncropped scans of immunoblots.
