# Supplementary material for: Induced cortical tension restores functional junctions in adhesion-defective carcinoma cells
Source: Nat Commun. 2017 Nov 28;8:1834. doi: 10.1038/s41467-017-01945-y (PMC5705652; doi:10.1038/s41467-017-01945-y)
Supplement: Supplementary file 3 — Description of Additional Supplementary Files [file 41467_2017_1945_MOESM3_ESM.pdf]

Description of Additional Supplementary Files

File Name: Supplementary Movie 1
Description: Morphological changes of HT29 cells after nocodazole treatment.
Nocodazole (20 μM) was added immediately before the first frame. Frames were taken every 1 min for 95 min. Frame rate, 12 frames/s.

File Name: Supplementary Movie 2
Description: HT29 cells cultured in the absence (left) or presence (right) of nocodazole for a prolonged period.
Nocodazole (10 μM) was added to the culture immediately after the onset of image acquisition. Cyan and yellow arrows show examples of rounded cells and their daughter cells, respectively. Daughter cells never appear in nocodazole-treated cells. Frames were taken every 5 min for 24 hrs. Frame rate, 20 frames/s.

File Name: Supplementary Movie 3
Description: Dynamics of myosin light chain after nocodazole treatment.
Nocodazole (10 μM) was added to the culture of HT29 cells expressing MLC-EGFP after the onset of image acquisition, as indicated within the Video. Video focuses on the apical portions of the cells. Frames were taken every 2 min for 64 min. Frame rate, 12 frames/s.

File Name: Supplementary Movie 4
Description: Dynamics of F-actin in control HT29 cells.
Nocodazole (10 μM) was added to the culture of HT29 cells expressing Lifeact-EGFP after the onset of image acquisition, as indicated within the Video. Yellow asterisks indicate neighboring cells. Videos focus on the apical portions of the cells. Frames were taken every 2 min for 64 min. Frame rate,12 frames/s.

File Name: Supplementary Movie 5
Description: Dynamics of F-actin in αE-catenin–depleted HT29 cells.
Nocodazole (10 μM) was added to the culture of αE-catenin–depleted HT29 cells expressing Lifeact-EGFP after the onset of image acquisition, as indicated within the Video. Yellow asterisks indicate neighboring cells. Videos focus on the apical portions of the cells. Frames were taken every 2 min for 64 min. Frame rate,12 frames/s.

File Name: Supplementary Movie 6
Description: Laser ablation of actin-based protrusions.
UV laser was applied to a Lifeact-EGFP–labeled protrusion at the point indicated by yellow arrowhead. HT29 cells were treated for 1 hr with 10 μM nocodazole prior to image acquisition. Frames were taken every 1 sec for 10 sec. Frame rate,12 frames/s.

File Name: Supplementary Movie 7
Description: Laser ablation of actin-based protrusions in vinculin–depleted HT29 cells. UV laser was applied to a Lifeact-EGFP–labeled protrusion at the point indicated by yellow arrowhead. Vinculin–depleted HT29 cells were treated for 1 hr with 10 μM nocodazole prior to image acquisition. Frames were taken every 1 sec for 10 sec. Frame rate,12 frames/s.

File Name: Supplementary Data 1
Description: Chemical compounds that depolymerize microtubules and simultaneously promote apical junction formation. The numbers in parentheses represent the hit number for each compound.
